# Crows protect visual working memory against interference

**DOI:** 10.1242/jeb.245453

**Published:** 2023-02-28

**Authors:** Lysann Wagener, Paul Rinnert, Lena Veit, Andreas Nieder

**Affiliations:** ^1^ Animal Physiology, Institute of Neurobiology, University of Tübingen, 72076 Tübingen, Germany; ^2^ Neurobiology of Vocal Communication, Institute of Neurobiology, University of Tübingen, 72076 Tübingen, Germany

**Keywords:** Corvid songbird, Visual working memory, Distractor resistance

## Abstract

Working memory, the ability to actively maintain and manipulate information across time, is key to intelligent behavior. Because of the limited capacity of working memory, relevant information needs to be protected against distracting representations. Whether birds can resist distractors and safeguard memorized relevant information is unclear. We trained carrion crows in a delayed match-to-sample task to memorize an image while resisting other, interfering stimuli. We found that the repetition of the sample stimulus during the memory delay improved performance accuracy and accelerated reaction time relative to a reference condition with a neutral interfering stimulus. In contrast, the presentation of the image that constituted the subsequent non-match test stimulus mildly weakened performance. However, the crows' robust performance in this most demanding distractor condition indicates that sample information was actively protected from being overwritten by the distractor. These data show that crows can cognitively control and safeguard behaviorally relevant working memory contents.

## Teaser

**Summary:** Crows possess active working memory enabling them to cognitively control and safeguard the memorization of relevant information.

## INTRODUCTION

The maintenance of information over brief delay periods can be achieved by different cognitive systems (Shevlin, 2020). In the case of simple short-term memory (such as iconic and echoic memory), a stimulus trace is temporarily retained in a passive, implicit way; short-term memory is fragile and highly susceptible to erasing by a successive stimulus. In contrast, working memory addresses a system by which the memory contents depend on attention and can be held and manipulated towards a goal in an active, explicit state; for as long as attention is directed at memorized relevant information, it can be protected not only from passive decline but also from interfering irrelevant stimuli (Luck and Vogel, 1997; Cowan, 2008; Baddeley, 2012; Carruthers, 2013; Oberauer, 2019; Nieder, 2022).

When exploring memory capacities in animals, this distinction is crucial. Animals are typically tested in variations of ‘delayed response tasks’ that contain a brief temporal gap between a stimulus and a response. However, an animal's success in a delayed response task does not yet indicate working memory because passive short-term memory typically suffices to explain performance (Nieder, 2022). One way to segregate passive short-term memory from active working memory is the presentation of interfering stimuli during memory retention. With only passive short-term memory at work, memory performance suffers greatly after distraction (Scott et al., 2012). However, with working memory capabilities, animals are able to largely ignore and filter out distracting information (Jacob and Nieder, 2014). Of course, animals – and corvids in particular – can store information for much longer durations in long-term memory (Kamil et al., 1994; Balda and Kamil, 1989; Olson, 1991; Olson et al., 1995; Gould-Beierle, 2000); however, to access this information from long-term memory, it needs to be retrieved into working memory.

**Fig. 1.**
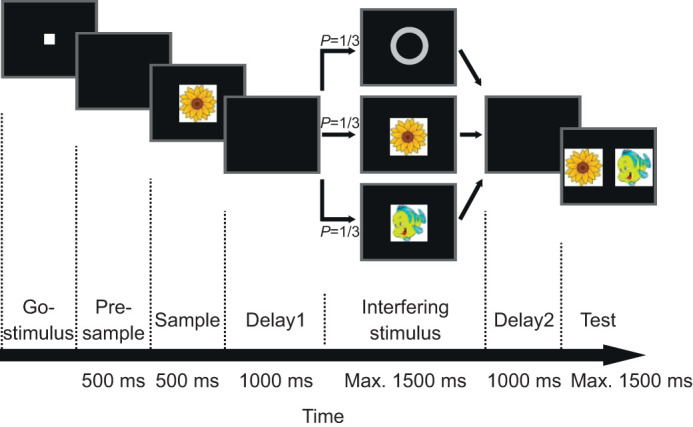
**Schematic illustration of the delayed match-to-sample task with interfering stimuli.** In each trial sequence (left to right), one of three interfering stimuli was shown halfway through the delay period: a neutral gray circle, repetition of the sample stimulus, or an image that served as the non-match in the impending test period. The crows needed to peck at the interfering stimuli to continue the trial. The crows indicated their choice in the test period by pecking at the selected test image (here, the flower would be the correct match).

Several bird species have been tested successfully for their ability to memorize information across short temporal gaps (e.g. Blough, 1959; Roberts, 1980; Regolin et al., 2005; Veit et al., 2015; Rinnert et al., 2019; Rinnert and Nieder, 2021). The delayed match-to-sample (DMS) task is a suitable task to investigate memory capacities in animals (Hunter, 1913; Lind et al., 2015). In the DMS task, an animal is first presented with a sample stimulus that is afterwards removed. After a delay period in which no stimulus is displayed, two or more choice stimuli are presented. The subject receives a reward for selecting the one that matches the sample. Different species of birds, such as pigeons (Blough, 1959; Roberts, 1980; Johnston et al., 2019), chickens (Nakagawa et al., 2004), black-capped chickadees, dark-eyed juncos (Brodbeck and Shettleworth, 1995), jays (Olson et al., 1995) and carrion crows (Goto and Watanabe, 2009; Veit et al., 2014; Hartmann et al., 2018; Ditz and Nieder, 2016; 2020; Wagener and Nieder, 2017; 2020; Balakhonov and Rose, 2017) can master the DMS task. However, so far it is not known whether corvids or other birds can actively protect memorized information against interference as an essential feature of working memory. In the current study, we therefore modified the classic DMS task by introducing an interfering stimulus following the presentation of the sample halfway through the delay period (Fig. 1). To succeed in the face of distraction, the animals need to actively maintain relevant sample information and to safeguard it by filtering out distractors (Jacob and Nieder, 2014; Jacob et al., 2018).

We considered two hypotheses. Target representation in memory could deteriorate in the face of strong task-irrelevant distractors, indicating that crows rely primarily on interference-vulnerable and passive short-term memory. Alternatively, the crows' memory performance could remain largely unaffected by interfering information, suggesting active filtering and suppression of distractor information characteristic of explicit cognitive control of memory contents. We found clear evidence for the latter.

## MATERIALS AND METHODS

### Subjects

One 2 year old female and one 2 year old male carrion crow (*Corvus corone* Linnaeus 1758) were used in this study. The crows were housed in social groups in indoor aviaries. During the training and testing period, they were on a controlled feeding protocol. Food was given as a reward during the sessions. Water was available *ad libitum* in the aviary and during the experiments. All procedures were carried out according to the guidelines for animal experimentation and approved by the responsible national authorities, the Regierungspräsidium Tübingen, Germany.

### Experimental setup

The crows were placed on a perch in front of a touchscreen monitor (3M Microtouch, 15 inch, 60 Hz refresh rate) in a darkened operant conditioning chamber (length 1 m, width 0.76 m, height 1 m). The behavior was controlled by the CORTEX system (National Institute of Mental Health, Bethesda, MD, USA) which also stored the behavioral data. An automated feeder delivered either mealworms (*Tenebrio molitor* larvae) or bird seed pellets upon correctly completed trials. An infrared light barrier was installed above the crows' head to which a reflector foil was attached. Except for the distractor and test periods, the crow had to keep its head still within the beam of the light barrier and thereby in front of the touchscreen throughout a trial.

### Behavioral task

The crows were trained on a DMS task in which they matched images (Fig. 1). A crow started a trial by positioning its head in front of the monitor whenever a go-stimulus (a small white square) was shown on the screen. When the head was in the correct position in front of the monitor, the crows received auditory feedback. After a 500 ms pre-sample phase with no stimulus, the sample stimulus (500 ms duration) was displayed. Colorful complex images were used as stimuli.

The sample was followed by a 1000 ms delay period (delay1) with a blank screen. Next, one of three interfering stimuli was shown in equal trial proportions (one-third) and pseudo-randomly interleaved: a gray circle that was never shown in the sample or test periods (neutral-image trials), the initially shown sample image (repeat-sample trials) or the image that was shown as a non-match stimulus in the subsequent test period (distractor trials). To ensure that the crow was perceiving the interfering stimulus, it had to peck at it within 1500 ms to continue the trial, and thereafter to resume the correct head position in front of the monitor. After a second 1000 ms delay period (delay2), the test period displayed two choice images side by side. To receive a reward, the crow had to peck at the test stimulus that matched the sample (‘match’) within 1500 ms while ignoring the non-matching stimulus (‘non­-match’). Match and non-match were pseudo-randomly and equally often shown on the left or right side. For every session, three new sample and non-match images were selected. Responses to the non-match were considered as error and not rewarded. Premature head movements (except during the interfering stimulus and test period) ended the trial, which was then discarded. The tests began once the crows' accuracy reached at least 75% correct responses per session. Each session consisted of an average of 412 completed trials for crow 1 and 446 completed trials for crow 2.

### Data analysis

The percentage of correct responses, i.e. the number of correct trials divided by the total number of completed trials, was calculated as a measure of behavioral accuracy. As a second measure, the reaction time (RT), i.e. how quickly the crows pecked the correct match stimulus in the test phase, was calculated. Accuracy and RT were calculated separately for the three interfering stimulus conditions. The relationship between accuracy and RT was measured using Pearson correlation. MATLAB (version R2020b, MathWorks Inc., Natick, MA, USA) was used for all data analyses.

## RESULTS AND DISCUSSION

The two crows performed a modified version of a visual DMS task, in which a task-irrelevant, interfering image was presented halfway through the working memory period (Fig. 1). Sample and test images varied in individual trials so that the crows had to flexibly memorize what they saw on a trial-by-trial basis. To test the crows' working memory, one of three different types of ‘interfering stimuli’ was presented within the delay period: ‘neutral-image trials’ as a reference condition, ‘repeat-sample trials’ and ‘distractor trials’. The crows' performance in these three conditions was compared using percentage correct performance (performance accuracy) and RTs as quantitative parameters.

**Fig. 2.**
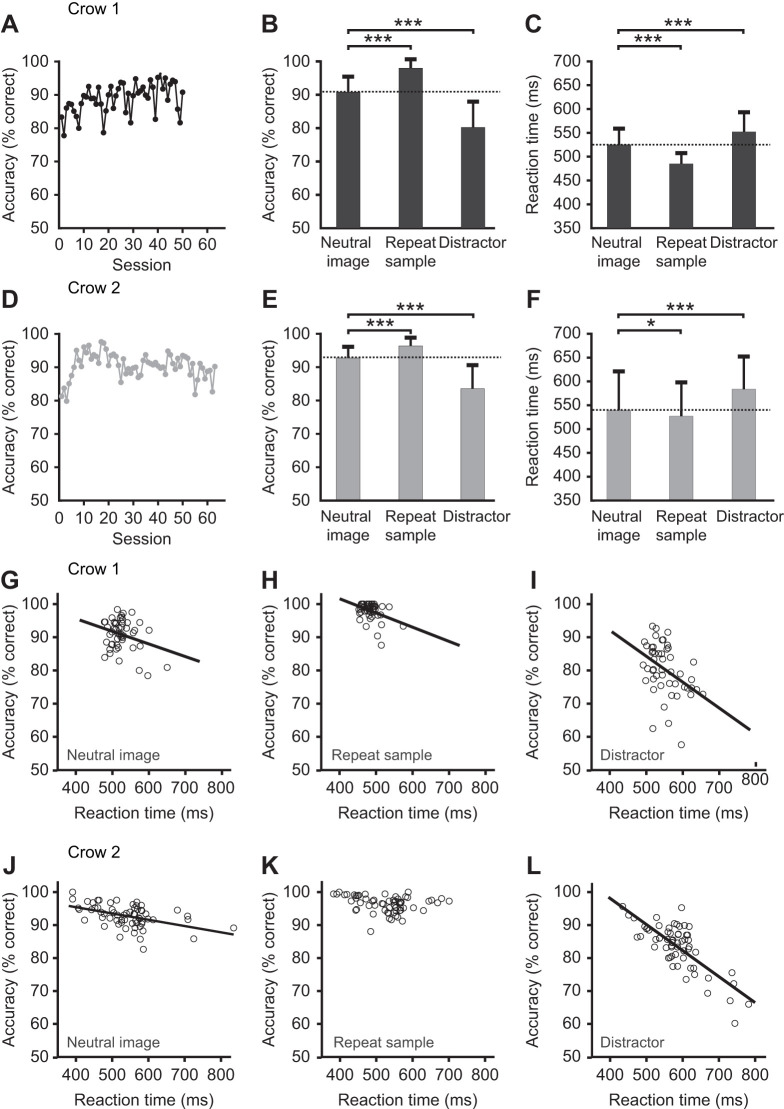
**Behavioral data.** (A) Accuracy (mean values) of crow 1 across sessions. (B) Average accuracy of crow 1 for the neutral, repeat-sample and distractor trials. Shown are the means across sessions and the s.d. The dotted line indicates the reference value for neutral trials. (C) Average reaction time of crow 1 for the neutral, repeat-sample, and distractor trials. Shown are the means across sessions and the s.d. The dotted line indicates the reference value for neutral trials. (D) Accuracy of crow 2 across sessions. (E) Average accuracy of crow 2 for the neutral, repeat-sample and distractor trials. Same layout as in B. (F) Average reaction time of crow 2 for the neutral, repeat-sample and distractor trials. Same layout as in C. (G–I) Correlation of accuracy with reaction time for neutral trials (G), repeat-sample trials (H) and distractor trials (I) across all sessions for crow 1. (J–L) Same as in G–I, but for crow 2.

Across all sessions, crow 1 reached a mean (±s.d.) performance accuracy of 88.9±4.5% (across 50 sessions), while crow 2 showed a mean accuracy of 90.4±3.8% (across 63 sessions) (Fig. 2A,D). The average accuracy of both crows with all three trial types in each daily session was significantly above the 50% chance level (each crow *P*<0.001, two-tailed binomial test). The crows' accuracy systematically differed between the three interfering stimulus conditions (each crow *P*<0.001, ANOVA) (Fig. 2B,E). Relative to the reference accuracy for the neutral-image trials (crow 1: 90.8±4.6%; crow 2: 92.8±3.3%), accuracy increased for repeat-sample trials (crow 1: 98.0±2.6%; crow 2: 96.4±2.5%; each crow *P*<0.001; paired-sample *t*-test, Bonferroni corrected) (Fig. 2B,E). In contrast, accuracy decreased relative to the neutral-image trials for both crows in distractor trials (crow 1: 80.2%±7.8%; crow 2: 83.6±7.0%; each crow *P*<0.001; paired-sample *t*-test, Bonferroni corrected) (Fig. 2B,E). Thus, across both crows, repetition of the sample during the memory delay enhanced accuracy on average by 5.25%, while the presentation of the non-match stimulus deteriorated accuracy by 9.9%.

As second performance parameter, we explored RT. Across all sessions, crow 1 had a mean (±s.d.) RT of 516.0±29.7 ms (across 50 sessions), while crow 2 showed a mean RT of 549.3±69.9 ms (across 63 sessions). The crows' RT systematically differed between the three interfering stimulus conditions (each crow *P*<0.001, ANOVA) (Fig. 2C,F). Relative to the reference RT for the neutral-image trials (crow 1: 525.0±33.9 ms; crow 2: 539.7±81.3 ms), RT decreased for repeat-sample trials (crow 1: 485.0±22.5 ms; crow 2: 527.0±70.9 ms; crow 1: *P*<0.001, crow 2: *P*<0.029; paired-sample *t*-test, Bonferroni corrected) (Fig. 2C,F). In contrast, RT increased relative to the neutral-image trials for both crows in distractor trials (crow 1: 552.1 ms±41.2 ms; crow 2: 583.7±68.6 ms; each crow *P*<0.001; paired-sample *t*-test, Bonferroni corrected) (Fig. 2C,F). Thus, repetition of the sample during the memory delay sped responses up by 26.4 ms on average, while presentation of the non-match stimulus slowed responses down by 35.6 ms across both crows.

The findings so far indicated an inverse relationship between accuracy and RT: more difficult conditions resulted in longer RTs. To systematically explore this relationship, we correlated accuracy and RT on a session-by-session basis. For each crow individually, we found a significant negative correlation of accuracy with RT (crow 1: *r*=−0.377, *P*=0.007; crow 2: *r*=−0.731, *P*<0.001; Pearson correlation). We tested this correlation for each of the three trial conditions and two crows separately. Significant negative correlations were found in crow 1 for all three conditions (neutral-image trials: *r*=−0.281, *P*=0.048; repeat-sample trials: *r*=−0.377, *P*=0.007; distractor trials: *r*=−0.415, *P*=0.003; *n*=50) (Fig. 2G–I). Similarly, significant negative correlations were found in crow 2 for neutral-image trials (*r*=−0.463, *P*<0.001; *n*=63) and distractor trials (*r*=−0.771, *P*<0.001) (Fig. 2J,L), whereas repeat-sample trials showed only a negative tendency (*r*=−0.239, *P*=0.061) (Fig. 2K). This confirms that performance accuracy and RT were negatively correlated irrespective of the interfering stimulus condition.

Our data show that the crows were affected by different types of interfering information during the delay period. Importantly, the crows managed surprisingly well to safeguard the relevant sample stimulus from demanding distraction. The results indicate that crows can actively protect relevant sample information from being erased by the distractor, thus emphasizing the crows’ cognitive aptitude (Nieder, 2017). Crows possess active working memory, enabling them to cognitively control the memorization of relevant information. Whether interfering information during delay times longer than the 3 s used in the current study would elicit comparable effects remains to be seen.

To guarantee that the interfering stimuli were perceived, we required the crows to peck at them. This constraint prevented us from testing performance without any interfering stimulus as a reference situation, as it would have lacked a motor response that alone could explain potential differentiating effects compared with interfering stimulus conditions. Instead, we used the neutral-image condition with a simple circle as a performance reference. In preceding pilot tests, a circle as the interfering stimulus was found to elicit indifferent accuracy and RT performance compared with no interfering stimulus in crow 1.

Relative to the neutral-image condition, significant improvement in performance (in terms of both accuracy and RT) was found for the repeat-sample trials in both crows. This finding suggests that the crows benefitted from repeating the relevant sample information in working memory to achieve higher performance. Such maintenance of relevant information by repetition in working memory is captured in Baddeley's working memory model (Baddeley, 2003).

Relative to the neutral-image condition, a mild but significant decay in performance (in terms of both accuracy and RT) was found for the distractor trials with the non-match stimulus as an interfering stimulus in both crows. Distractor trials were certainly the most difficult condition because not only did the non-match stimulus belong to the same complex picture category as the match, a situation known to elicited the highest distraction (Yoon et al., 2006; Sreenivasan and Jha, 2007), but also the distractor was the only other response option besides the match in the test phase. The crows' continued high performance in this distractor condition clearly indicates that sample information was actively protected and cognitively controlled from being overwritten by the distractor. It seems crows can attenuate the processing of distracting information due to endogenous attentional biasing towards relevant sample information during working memory maintenance (Quest et al., 2022). At the same time, more frequent selection of the distractor in the test phase (and thus more errors) also signifies that the distractor was not eliminated but held in memory. These findings suggest that crows can maintain more than one item at a time in working memory. This has also been suggested for visual change detection tasks in humans, pigeons and crows (Gibson et al., 2011; Balakhonov and Rose, 2017).

In both crows and across interfering stimulus conditions, performance accuracy and RTs were negatively correlated. Thus, higher RTs were associated with higher error rates. That trials with longer RTs are more likely to be errors has also been widely reported for perceptual decision making in humans and other primates when task difficulty is fixed (Carter et al., 1998; Shevinsky and Reinagel, 2019). Only rats show a positive correlation (Shevinsky and Reinagel, 2019). In that respect, crows tend toward producing a more primate-like behavioral pattern.

Why are interfering stimuli not entirely suppressed or filtered out? In the ecological environment of an animal, any stimulus could potentially contain relevant information, maybe even more important information than the task at hand (Berti and Schröger, 2003). It would therefore be maladaptive to completely ignore interfering stimuli. The ‘supervisory attentional system’ has to react to unexpected and potentially meaningful stimuli to be adaptive (Norman and Shallice, 1986). Working memory is able to coordinate the maintenance of distractibility and the focus on the task at hand; the more difficult and attention-demanding a memory task, the less distraction is seen (Berti and Schröger, 2003).
